# Clinical Cases

**Published:** 1887-12

**Authors:** J. A. Biegler

**Affiliations:** Rochester, N. Y.


### CLINICAL CASES.

J. A. Biegler, M. D., Rochester, N. Y.

Sanguinaria in Follicular Angina, Tonsillitis, Diphtheritis: Geo. H., age, seventeen years, scrofulous diathesis. From infancy breathed with his mouth open, especially during sleep. In later years he was more or less a subject for the above-named affections, frequently the latter. Two sisters died in infancy of diphtheria. On the last occasion of my attendance, six or seven years ago, for diphtheria, he had the following symptoms: Intense heat and dryness of the throat, amounting to a burning sensation; choking feeling when swallowing; *pearly* coating on fauces and tonsils, worse on right side; tonsil on that side most inflamed. The general symptoms were not recorded, but the sickness was of the grade of diphtheria, not of catarrh.

One dose of Sanguinaria$^{cm}$ was given, and then I said, "If this is the remedy for this attack, he will not have another," and I simply state the fact that this result has been realized, as he has not had one since that time, notwithstanding that I have been called more than twenty times in that period to attend the sisters and mother for similar attacks.

PRURITUS: Case —. A lady, seventy-two years old, had been tormented to the end of endurance, and had now become desperate by suffering from this disease. Itching was so intense WHEN LYING DOWN that she was forced to sit night and day. PARTS SWOLLEN AND BULGING. September 21st, 1886, she received four powders of Collinsonia$^{cm}$, to take every four days. She was much relieved in a few days, and cured before the remedy was all taken. (See Eggert, page 390.)

This patient returned October 22d, complaining of soreness of the external meatus and a burning sensation when urinating, which was relieved by BATHING IN COLD WATER. For this she received two doses of Pulsatilla$^{cm}$, one dose at once, the other in five days, if necessary, but which was not taken, as the trouble was cured before that time. This is another case for which one of the gentry of the class I have heretofore described had for weeks treated this sufferer by mimicking his superiors of the old school by dabbling her with all kinds of washes—one of that class who, for their nefarious purpose, PUBLICLY profess and defend Homœopathy, and, with the hope of prolonging their deceitful career, explain that the materia medica is still so limited in its resources that they are compelled to give several remedies at the same time, or in alternation, to obtain the result that the right remedy, which THEY have not yet discovered, would accomplish; also to supply the deficiency by a resort to adjuvants, etc., "you know," stolen from the antiquated books of old physic, which they parade as the outcome of their scientific noddles. This is the class that will enjoy millennial happiness when Arndt & Co. have succeeded in reconstructing the work of Hahnemann and his followers to the plane of their understanding.

SCIATIC RHEUMATISM: Case —. A man over seventy years old suffered from this disease several years, during which time he obtained no relief, although under the care of a homœopathist in the last two years.

April 26th, 1887, complained of pain in the left hip and thigh. *Worse in stormy, wet weather, from sitting long, and when lying on the opposite side. Relief from walking or motion and from warmth.* Weak, gone feeling in the stomach before meals. Milk disagrees. Gave one dose of Rhus tox.$^{mm}$.

May 6th, pain in hip and thigh better, notwithstanding a severe cold, from which he now suffered. The present symptoms are chilliness in the back, chill runs upward; pain in left thigh is a burning pain (see Hering's *Condensed Materia Medica*). Gave Lachesis$^{cm}$, one dose.

May 13th, comes in happy; says he has not been so well in years; continues so at this date. Simple as this case is, the result ought to be an every-day experience, but it is not so, and it is, therefore, selected as contrast to the treatment and results obtained by the gentlemen who prate like parrots about the law of cure, and, with a like dignified composure and capacity to make indistinct their meaning (if they have any), declare to the world their ability to supersede that law, and, whenever it fails them in the cure of disease, that they with their wisdom are ready to fill the void with hypodermic injections, Chloroform, and Aconite liniments, and with the "usual homœopathic remedies."—*Clinical Bureau International Hahnemannian Association.*

